# Accuracy Analysis of 3D Bone Fracture Models: Effects of Computed Tomography (CT) Imaging and Image Segmentation

**DOI:** 10.1007/s10278-024-00998-y

**Published:** 2024-03-14

**Authors:** Martin Bittner-Frank, Andreas Strassl, Ewald Unger, Lena Hirtler, Barbara Eckhart, Markus Koenigshofer, Alexander Stoegner, Arastoo Nia, Domenik Popp, Franz Kainberger, Reinhard Windhager, Francesco Moscato, Emir Benca

**Affiliations:** 1 Department of Orthopedics and Trauma Surgery, Medical University of Vienna, Währinger Gürtel 18-20, 1090 Vienna, Austria; 2 Center for Medical Physics and Biomedical Engineering, Medical University of Vienna, Vienna, Austria; 3 Department of Biomedical Imaging and Image-guided Therapy, Medical University of Vienna, Vienna, Austria; 4 Center for Anatomy and Cell Biology, Medical University of Vienna, Vienna, Austria; 5 Ludwig Boltzmann Institute for Cardiovascular Research, Vienna, Austria; 6 Austrian Cluster for Tissue Regeneration, Vienna, Austria

**Keywords:** Pre-operative planning, 3D model, Bone fracture, Trauma surgery, Accuracy assessment

## Abstract

The introduction of three-dimensional (3D) printed anatomical models has garnered interest in pre-operative planning, especially in orthopedic and trauma surgery. Identifying potential error sources and quantifying their effect on the model dimensional accuracy are crucial for the applicability and reliability of such models. In this study, twenty radii were extracted from anatomic forearm specimens and subjected to osteotomy to simulate a defined fracture of the distal radius (Colles’ fracture). Various factors, including two different computed tomography (CT) technologies (energy-integrating detector (EID) and photon-counting detector (PCD)), four different CT scanners, two scan protocols (i.e., routine and high dosage), two different scan orientations, as well as two segmentation algorithms were considered to determine their effect on 3D model accuracy. Ground truth was established using 3D reconstructions of surface scans of the physical specimens. Results indicated that all investigated variables significantly impacted the 3D model accuracy (*p* < 0.001). However, the mean absolute deviation fell within the range of 0.03 ± 0.20 to 0.32 ± 0.23 mm, well below the 0.5 mm threshold necessary for pre-operative planning. Intra- and inter-operator variability demonstrated fair to excellent agreement for 3D model accuracy, with an intra-class correlation (ICC) of 0.43 to 0.92. This systematic investigation displayed dimensional deviations in the magnitude of sub-voxel imaging resolution for all variables. Major pitfalls included missed or overestimated bone regions during the segmentation process, necessitating additional manual editing of 3D models. In conclusion, this study demonstrates that 3D bone fracture models can be obtained with clinical routine scanners and scan protocols, utilizing a simple global segmentation threshold, thereby providing an accurate and reliable tool for pre-operative planning.

**Supplementary Information:**

The online version contains supplementary material available at 10.1007/s10278-024-00998-y.

## Introduction

Three-dimensional (3D) printing is gaining increasing interest in pre-operative planning, especially in orthopedic and trauma surgery [1, 2]. Among the major advantages are a reduced operation time [3, 4] and radiation exposure [3, 5], in addition to an improved patient interaction in explanation of the surgery [6, 7]. However, additional costs and time are needed for 3D model design and manufacturing [4], limiting its frequent use in clinical routine [8]. Particularly trauma surgery might benefit from 3D-printed models in pre-operative planning for bone fracture treatment. Their use provides several advantages, such as giving tactile feedback [5, 9, 10] (especially important in articular fractures [11]), selection of better fitting osteosynthesis plates [3, 12–14] and their pre-bending [3, 15–18]. Furthermore, the intra-operative use of 3D printed osteotomy guides (e.g., for corrective osteotomy [17, 19–22]) and anatomical models [3] (as a reference) also enable a higher accuracy than conventional surgery. Sequentially, 3D printed models have been previously successfully used in the treatment of distal radius [7, 13], scaphoid [16], tibia plateau [3], and acetabulum fractures [9, 15, 23]. However, available studies are mostly limited to single-case or case series reports. No systematic investigation of the inherent errors and their effect on 3D model accuracy has been performed so far. This knowledge gap will likely limit the usability of 3D printed models due to the time- and cost inefficiency when developing 3D models without a defined workflow. Identifying error sources and quantifying their effect will allow to establish an optimized workflow for 3D model design and their highest dimensional accuracy.

3D models rely on a 3D imaging modality, including CT imaging. These imaging sequences are a digital reconstruction of assessed anatomy but do not necessarily represent the true anatomy. Thus, errors in 3D models are potentially attributed to CT images (image acquisition and reconstruction), image processing (segmentation, triangulation, and post-processing), but also the 3D printing process [24]. Previously, significant differences in model accuracy were determined for selected CT scanner types [25], slice thicknesses [26], reconstruction kernels [27], segmentation algorithms [24, 28, 29], and triangulations [30], whereby segmentation was considered to cause the highest dimensional inaccuracies [24]. In contrast, a comparison of 3D models generated from low-dosage CT protocols versus clinical protocols indicated no difference in model accuracy [25, 31]. A limitation of those studies is their focus on a single variable within an entire workflow for 3D-printed anatomical model design. Further, they are typically performed on intact bone specimens and do not account for the potential effect of a fracture on the accuracy of 3D bone models. Recently, photon-counting detector (PCD) CT has evolved as a novel technology for clinical applications with an increased spatial resolution of bony details, in comparison to conventional energy-integrating detector (EID) CTs [32]. However, no investigation on the quality of obtained 3D bone fracture models for 3D printing for different CT detector technologies has been performed yet.

The focus of this study was to determine the errors attributed to different CT technologies/scanners, scan protocols (clinical versus high dosage for increased model accuracy), segmentation algorithms, as well as specimen orientation during CT scanning and consequently quantify the resulting dimensional deviations on a defined bone fracture model. Errors attributed to the 3D printing process itself were neglected since they are considered to be small (< 0.1 mm) [24, 29, 33] and clinically irrelevant. Hence, the proposed work aimed to (a) introduce a specific clinically common bone fracture model, (b) establish a defined workflow for CT image acquisition and image processing, and finally (c) quantify the effect on the accuracy in all processing steps. In the current study, the distal radius fracture (Colles’ fracture) was selected as a model for multiple reasons. It is among the most common fractures [34], the average location of the fracture line has been accurately described [35], and articular fractures have already been used for pre-operative planning [7, 13]. It was hypothesized that there was a significant effect of the investigated variables on 3D model accuracy considering the maximal acceptable deviation in clinical routine.

## Materials and Methods

### Specimens

Paired anatomic forearm specimens were obtained from 10 body donors (5 male and 5 female, mean age 78 ± 8 years), provided by the Center for Anatomy and Cell Biology, Medical University of Vienna (study approved by the Ethics Committee of the Medical University of Vienna (EK-Nr: 2003/2019)). Forearm specimens were amputated at the midsection of the radius and ulna, fixed with two laces onto a plastic grid, and stored in air-tight plastic containers at − 20 °C until further usage.

### Fracture Preparation with 3D-printed Osteotomy Guides

**Fig. 1:**
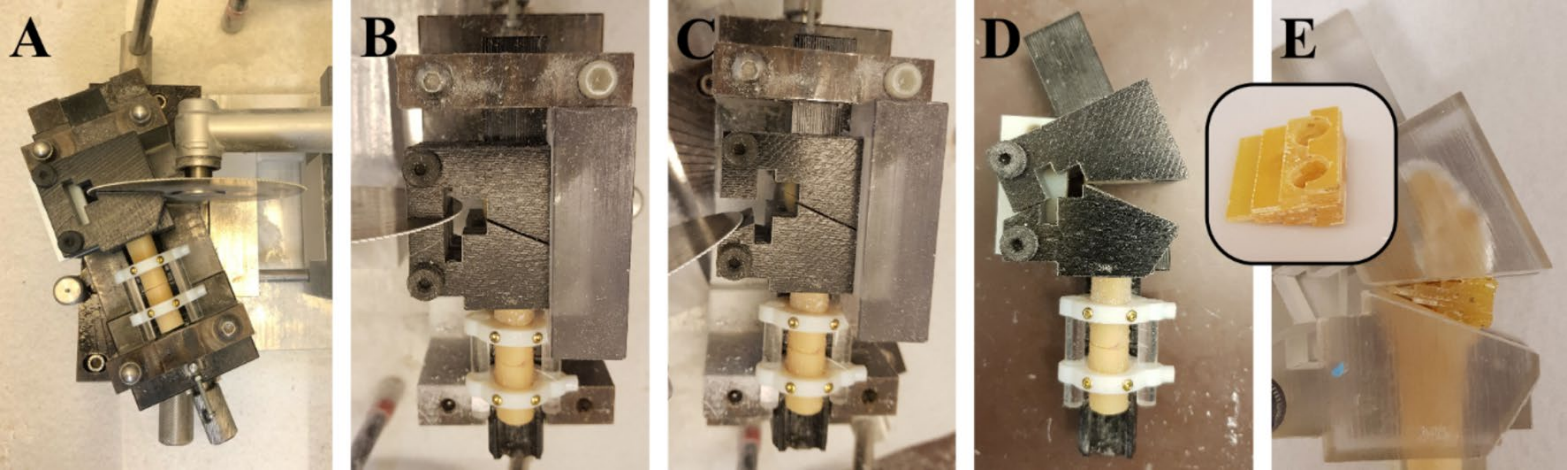
Manufacturing of a defined distal radius fracture with a 3D printed osteotomy guide. **A**: Cut with a buzz saw at a defined fracture line. **B** & **C**: Cut of the osteotomy guide. **D**: Dorsal tilting by 26.1°. **E**: Fixation with a wedge (see inset)

Forearm specimens were thawed for 24 h at 4 °C. Radii were extracted using a modified Henry approach, whereby all ligaments, the interosseus membrane, and the articular capsule were incised. Care was taken to minimize damage to surrounding soft tissue, as the forearm specimens were used to re-implant the radii following their fracture simulation. The explanted radii were macerated in water for two weeks at 60 °C to fully remove soft tissue. Specimen-specific osteotomy guides were designed in Mimics Research (V21.0; Materialise NV, Leuven, Belgium) to enable defined cutting and tilting of the distal radius, to mimic a loco typico fracture. Hereby, the fracture line was defined as previously described by Baumbach et al. [35]. In brief, the authors determined the fracture location on the dorsal and palmar side from radiographs of 157 dorsally displaced distal radius fractures in relation to the corresponding apices of the lunar facet. According to existing literature [35–38], the average volar inclination of the distal radius is 11.0° in its anatomic position, whereas Baumbach et al. [35] measured a volar inclination of -15.1° on dorsally displaced distal radius fractures. Hence, in the present study, the osteotomy guides were designed to enable a defined dorsal tilting of the distal fracture fragment by 26.1° from its anatomical position. Figure 1 illustrates sequential steps of the procedure: First, the osteotomy was executed on the radius specimens with a buzz saw at the predefined location. Second, the osteotomy guide was cut to enable its manipulation and dorsal inclination of the distal fracture fragment. The fracture fragment was secured by inserting and affixing a wedge into the fracture gap with super glue (Loctite 1,462,904, Henkel AG & Co. KGaA, Düsseldorf, Germany). The wedge, composedof five epoxy glass fiber plates milled using computerized numerical control (CNC), was affixed in a staggered arrangement. Precise positioning was achieved by incorporating two 6.0 mm fitting dowel pins (see inset in Fig. 1). Pilot CT scans confirmed the distinct contrast between epoxy glass fiber plates and bone tissue, facilitating their straightforward isolation in image-processing.

### 3D Surface Scanning

The surfaces of the fractured radius specimens underwent scanning using a high-resolution 3D scanning system (SmartSCAN HE-C8-8MP, Hexagon AB, Stockholm, Sweden; field of view: 250 mm, resolution: 64 μm (x and y direction), feature accuracy: 14 μm). Given the high resolution, 3D computer-aided design (CAD) models, generated from the surface scans, served as the ground truth for determining the dimensional accuracy of the CT-based 3D models. As radius specimens exhibited porosities on the surface and a thin shell in the epiphysis, a white powder (Crick 130, CRC 20,790-AJ, CRC Industries, Horsham Township, Pennsylvania, USA) was applied to enhance surface quality during scanning. The resulting surface models were exported as standard triangle language (STL) files and imported into 3-matic Research (V 13.0; Materialise NV, Leuven, Belgium). The epoxy glass wedge was manually removed and distinct epiphyseal and diaphyseal parts were created using the “trim” tool (in the following referred as surface scan models).

### CT Imaging and Processing

Following surface scanning, the fractured radius specimens were re-implanted, carefully approximating their original anatomical position inside the forearms. To secure the radii, the subcutaneous tissue was sutured (V13H, Vicryl, 3 − 0, Ethicon, Raritan, NJ, USA), and the skin was closed with cutaneous sutures (EthilonII, polyamide blue monofil 4 − 0, Ethicon, Raritan, NJ, USA). The subsequent CT scans of the forearm specimens were conducted using two different CT technologies and a total of four scanners. These included one PCD-CT (NAEOTOM Alpha) and three EID-CTs: SOMATOM Force, SOMATOM Edge Plus (all three: Siemens Healthineers AG, Forchheim, Germany), and Diamond Select Brilliance 64 CT (Koninklijke Philips N.V., Amsterdam, The Netherlands). For each scanner, two scanning protocols were employed: a clinical routine, as well as an optimized scan protocol. The routine protocol, utilizing a tube voltage of 120 kVp, aligns with the manufacturers’ protocols for bone imaging and is routinely applied at the authors’ institution. The optimized protocol aimed for higher image quality compared to clinical routine protocols typically used for diagnostic purposes in cases of suspected distal radius fractures. The corresponding protocols were standardized across all scanners (see Table 1), as far as equalization was feasible due to the usage of different scanner generations (device age), manufacturers, and potentially different scanner properties. Forearm specimens were positioned in their axial direction (longitudinally), and further rotated by 90° (transversal scanning rotation) for the SOMATOM Force using the routine protocol. This variation in patient positioning aimed to explore its impact on the accuracy of 3D models.

**Table 1:** CT scan protocols and reconstruction settings

**Scanner**	**Alpha**		**Force**		**Edge+**		**Brilliance**	
**CT technology**	**PCD**		**EID**		**EID**		**EID**	
**Scan protocol**	**Optimized**	**Routine**	**Optimized**	**Routine**	**Optimized**	**Routine**	**Optimized**	**Routine**
**Scan settings**								
Collimation in mm	120 × 0.2	120 × 0.2	64 × 0.6	64 × 0.6	64 × 0.6	64 × 0.6	64 × 0.625	64 × 0.625
Voltage in kVp	120	120	120	120	120	120	120	120
TCTP^*^ in mAs	250	55	350	76	350	85	300	80
Rotation time in s	0.5	0.5	1	1	1	1	1	1
Pitch factor	0.80	0.80	0.20	0.85	0.35	0.85	0.20	0.39
Mean CTDI in mGy	20.1	4.4	19.9	4.4	23.7	5.8	26.5	5.3
Mean DLP in mGycm	230	50	490	114	594	152	668	150
**Reconstruction settings**								
Slice thickness in mm	0.20	0.20	0.40	0.40	0.50	0.50	0.67	0.67
Increment in mm	0.20	0.20	0.40	0.40	0.50	0.50	0.67	0.67

*Tube Current Time Product

**Fig. 2:**
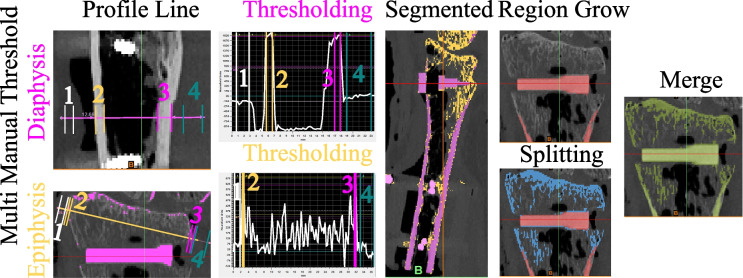
Segmentation with the multi manual threshold. A profile line is drawn in the center of the diaphysis and the epiphysis. Four regions are marked: **1** & **4**: soft tissue, **2** & **3**: cortical bone. Thresholding is performed as the 50% difference between those regions. The epi- and diaphysis are separated with region grow. The epiphysis is split to remove bone regions already present in the diaphysis and finally merged into one bone model

Image processing was conducted using Mimics Research (V21.0, Materialise NV, Leuven, Belgium). Mimics Research is well-established for evaluationing anatomical 3D models and was approved by the Federal Drug Administration (FDA) for medical applications [39], ensuring optimal comparability with previous studies and useability in future research. The segmentation of all CT image series employed a single automatic threshold (SAT) with a minimum set at 226 Hounsfield units (HU) and the radius models were isolated using the “region grow” tool. Manual removal of connecting voxels to the carpal bone was performed (using the multi-slice edit tool). Subsequently, the models were filled using the “smart fill” tool, considering that only the outer contour could be compared to the surface scans. Following this, 3D parts (CAD models) were generated (setting: “optimal”) and post-processed with the “wrap” (one pixel “smallest detail”, half a pixel as “gap closing distance”) and “smooth” tools (two iterations, smooth factor 0.3, according to [30]). Additionally, scan series from the SOMATOM Force utilizing the optimized protocol were segmented with a multi-manual threshold (MMT), as outlined in Fig. 2. In essence, specific thresholds for the cortex of the diaphysis and the epiphysis were determined using a line intensity profile. Four regions are defined in a single plane: soft tissue, bone cortex, bone cortex, and soft tissue and their HU values were evaluated. The lower segmentation threshold was set at a 50% difference between the assessed HU values, as described previously [40]. The upper segmentation value was not limited. Overlapping regions of the mask for the epiphysis were deleted using the “split” tool and then merged to obtain a single segmented mask. 3D part generation and post-processing were conducted analogouslyto the described SAT algorithm.

### Image Registration and Part Comparison Analysis

**Fig. 3:**
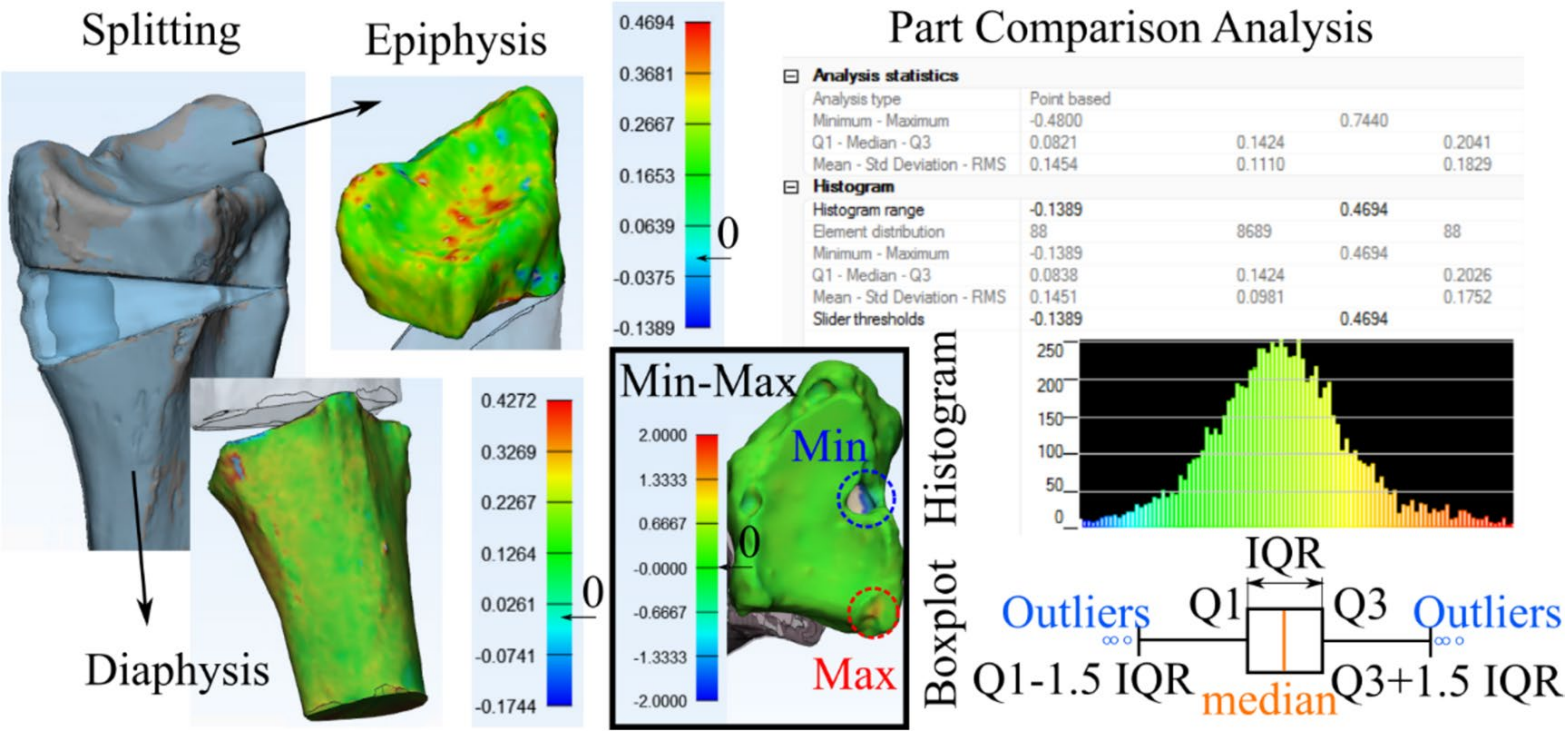
Part comparison analysis (PCA): The epoxy glass wedge is removed from the surface scan model, splitting radii into epiphysis and diaphysis. CT-models are registered onto surface scan models and PCA is performed for both regions separately, reported as histograms and boxplots (Q1: 1st quartile, Q3: 3rd quartile, IQR: inter-quartile range). Inset (Min-Max): Demonstration of elevated minimum (missing regions in CT-based 3D models) and maximum areas (overestimation in CT-based 3D models)

Obtained 3D models were imported into 3-matic Research (V 13.0, Materialise NV, Leuven, Belgium). Next, models generated from CT image series (in following: CT-models) were registered onto the surface scan models with a “3-point registration”, selecting the dorsal tubercle, the styloid process, and the most proximal point of the interosseous margin. For convergence of registration, a “global registration” algorithm was executed additionally (distance threshold: 1.000, 20 iterations, sample percentage 100). Analogue to the models generated from the surface scans, the epoxy glass wedge was removed manually, thus, splitting the radius models into the epiphysis and diaphysis. Separate evaluations for the epiphysis and diaphysis were performed since it was recognized in a pilot trial that the segmented SAT models show a clear dimensional overestimation in the diaphysis, but not as pronounced in the epiphysis. Further, the fracture line was superimposed with the transition region of the epiphyseal and diaphyseal region in the MMT algorithm, making the splitting in these two regions even more intuitive. For both regions, a “part comparison analysis” (signed) was performed for each CT-model, with respect to the corresponding surface scan model (see Fig. 3). The density distribution histograms were exported as text files and evaluated with a customized Python script in Spyder (4.2.5, Pyhton 3.8, The Scientific Python Development Environment). Hereby, the minimum (Min), maximum (Max), first quartile (Q1), median, third quartile (Q3), interquartile range (IQR), mean, standard deviation (std), and root mean square error (RMSE) were computed. Also, density distribution histograms, probability density functions (pdf, calculated with a kernel-density estimate using Gaussian kernels), and boxplots were plotted for data visualization.

### Inter- and Intra-operator Variability

Inter- and intra-operator variability were determined with respect to the MMT segmentation threshold for the epiphysis and diaphysis, and the 3D model accuracy (mean dimensional deviation of the SAT-based 3D model vs. the surface scan model). For both measures, CT scan series obtained with the optimal protocol of the SOMATOM Force scanner were used. Operator 1 (MBF, biomedical engineer with extensive experience in medical image segmentation, who developed the workflow and performed all evaluation steps) repeated the evaluations of the segmentation threshold and generation of the SAT-based 3D models after 8 weeks, to assess the intra-operator variability. Operators 2, 3, and 4 had different levels of experience in medical image segmentation and were all familiar with Materialise software package. Operator 5 had no experience and has never used the software before. Part comparison analyses performed individually by all five operators were used to determine the inter-operator variability.

### Statistical Analysis

Statistical analysis was performed in Scipy [41] (data from all 20 samples were pooled and evaluated for each investigated attribute, e.g. segmentation algorithm, causing ~ 100,000 data points for the dimensional deviations of the 3D surface nets). Density distribution histograms and the Kolmogorov-Smirnov test indicated a non-normal distribution of the majority of the data sets. Thus, the Mann–Whitney-U test (for two groups) and a Kruskal-Wallis test (for more than two groups) were used with a significance level of α = 0.05 to determine statistically significant differences in dimensions between the models. The p-values were adjusted with the Bonferroni correction for multiple testing. Hereby, testing was only performed between different 3D models for a direct comparison. The suitability of each model for clinical routine was determined by a simple criterion: if the dimensional mean deviation was below the required 0.5 mm.

Inter- and intra-operator variabilities were assessed in SPSS (v27, IBM Corporation, Armonk, New York, U.S.A.) by computing the intra-class correlation (ICC) with a two-way mixed model according to [42] at a significance level of α = 0.05. Hereby, values of ICC > 0.75 were rated as excellent, 0.74 > ICC > 0.60 as good, 0.59 > ICC > 0.40 as fair, and ICC < 0.39 as poor.

## Results

**Fig. 4:**
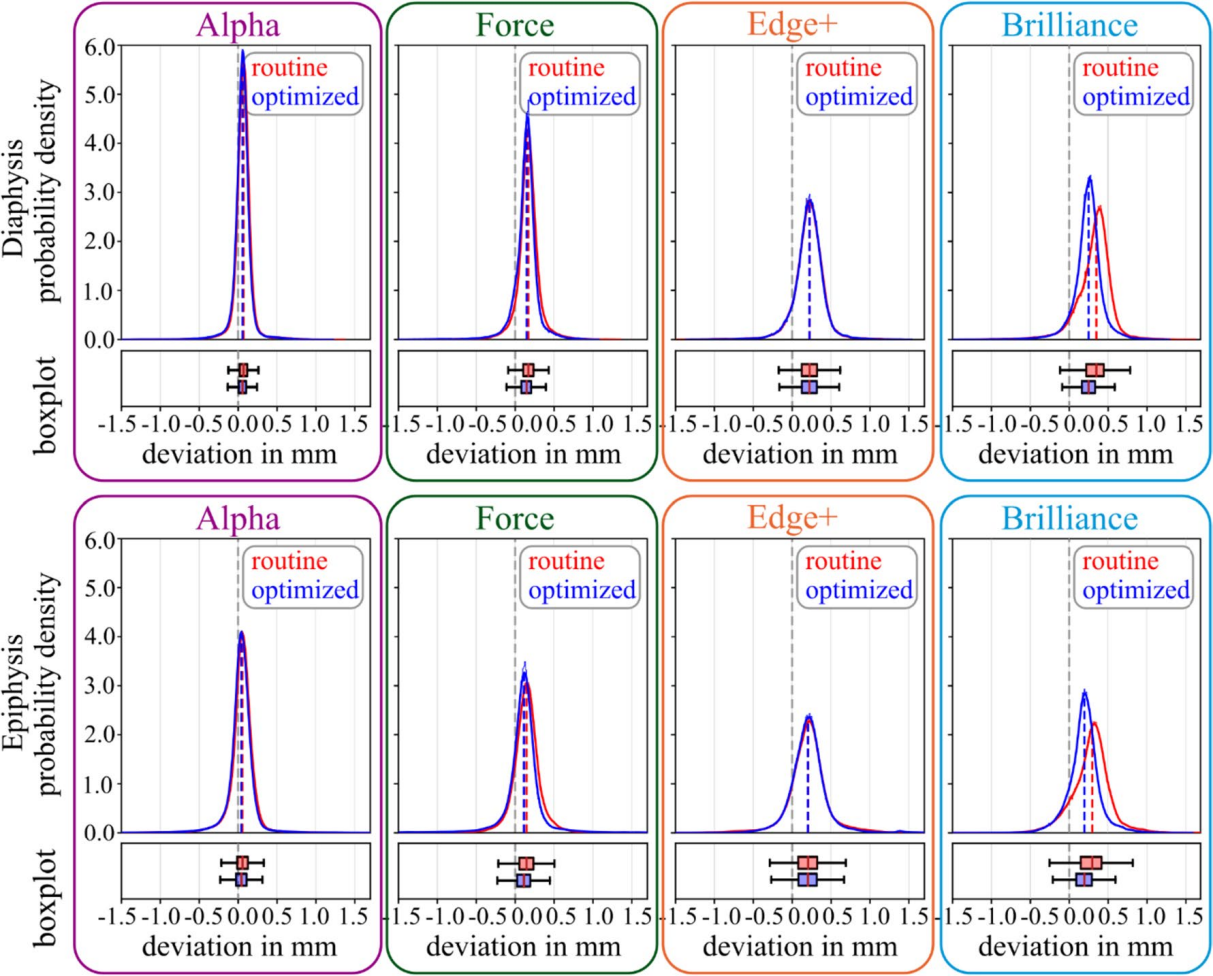
Deviation of 3D model dimensions, depending on CT technology/scanner and scan protocol. The top row indicates plots for the diaphysis, the bottom row for the epiphysis. Alpha, Force, Edge + and Brilliance are compared with respect to the routine and optimized scan protocol. Probability density functions are shown with histograms and corresponding boxplots (outliers are not plotted, for better visibility due to a large number of data points (∼100.000))

All investigated variables yielded CAD models with a mean dimensional deviation well below the required 0.5 mm for clinical routine. However, the selection of different CT technologies/scanners resulted in statistically significant (*p* < 0.001) differences in the accuracy of obtained CAD models (see Table 2; Fig. 4).

**Table 2:** Deviation of 3D model dimensions, depending on CT technology/scanner and scan protocol (routine and optimized, dia: diaphysis, epi: epiphysis, std: standard deviation, RMSE: root mean squared error, Min: minimum, Max: maximum, IQR: inter-quartile range; all units in mm)

	**Mean**	**Std**	**RMSE**	**Min**	**Max**	**Median**	**IQR**
**Alpha dia**							
Routine	0.07	0.13	0.15	-2.07	1.37	0.07	0.10
optimized	0.05	0.13	0.14	-1.99	1.22	0.06	0.09
**Alpha epi**							
Routine	0.04	0.20	0.20	-3.77	2.09	0.06	0.14
optimized	0.03	0.20	0.20	-3.04	2.12	0.04	0.14
**Force dia**							
Routine	0.17	0.15	0.23	-1.52	1.36	0.17	0.13
optimized	0.14	0.17	0.22	-2.02	1.07	0.15	0.13
**Force epi**							
Routine	0.14	0.22	0.26	-3.55	2.07	0.15	0.18
optimized	0.10	0.30	0.32	-3.94	2.15	0.11	0.17
**Edge + dia**							
Routine	0.22	0.20	0.30	-1.84	1.53	0.23	0.20
optimized	0.22	0.19	0.29	-1.36	1.54	0.23	0.19
**Edge + epi**							
Routine	0.19	0.30	0.35	-4.08	2.14	0.20	0.24
optimized	0.20	0.27	0.34	-3.65	2.08	0.20	0.23
**Brilliance dia**							
Routine	0.32	0.23	0.39	-1.96	1.64	0.35	0.23
optimized	0.24	0.19	0.31	-1.78	1.29	0.25	0.17
**Brilliance epi**							
Routine	0.28	0.26	0.38	-2.39	2.90	0.30	0.27
optimized	0.19	0.22	0.29	-1.92	1.60	0.19	0.20

**Fig. 5:**
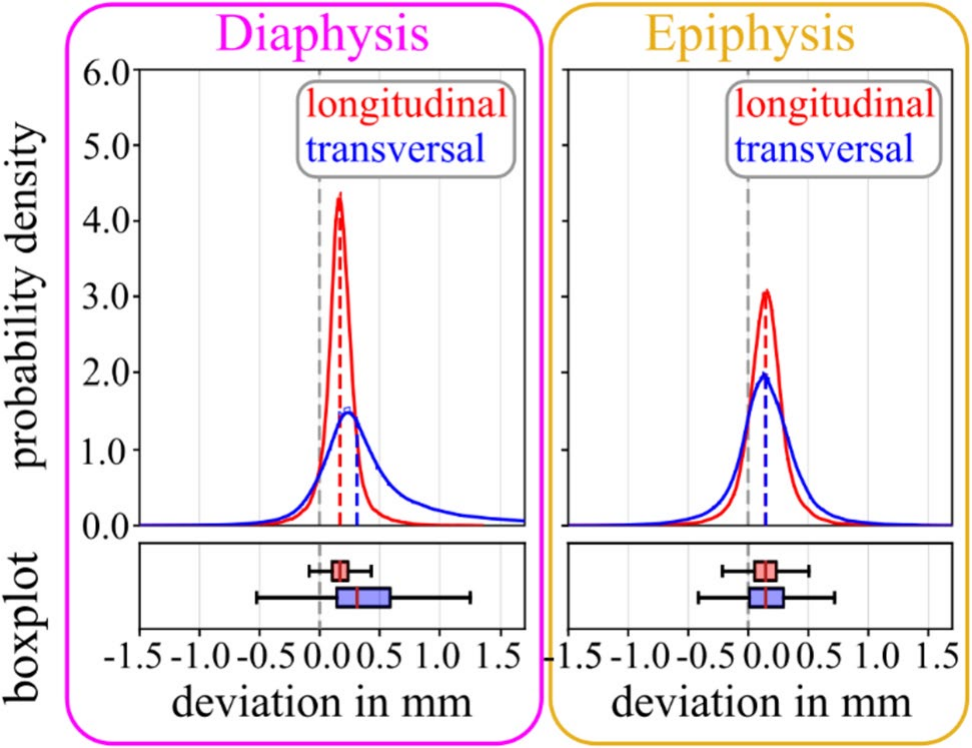
Deviation of 3D model dimensions, depending on scan direction (longitudinal vs. transversal), shown for diaphysis and epiphysis. Probability density functions are shown with histograms and corresponding boxplots (outliers are not plotted, for better visibility due to a large number of data points (∼100.000))

Further, 3D models obtained with the optimized protocol were more accurate than corresponding routine-based ones (for Alpha, Force, and Brilliance). However, no statistically significant difference (*p* > 0.05) could be observed between different protocols for the Edge + scanner. Orienting the specimens in the longitudinal direction of the forearm yielded significantly (*p* < 0.001) lower dimensional deviations of the corresponding 3D models of the diaphysis and epiphysis, compared to orienting specimens in transversal direction (see Table 3; Fig. 5).

**Table 3:** Deviation of 3D model dimensions, depending on specimen orientation (longitudinal and transversal, std: standard deviation, RMSE: root mean squared error, Min: minimum, Max: maximum, IQR: inter-quartile range; all units in mm)

	**Mean**	**Std**	**RMSE**	**Min**	**Max**	**Median**	**IQR**
**diaphysis**							
longitudinal	0.17	0.15	0.23	-1.52	1.36	0.17	0.13
transversal	0.45	0.56	0.71	-2.72	5.08	0.31	0.45
**epiphysis**							
longitudinal	0.14	0.22	0.26	-3.55	2.07	0.15	0.18
transversal	0.15	0.28	0.32	-1.72	2.17	0.15	0.29

Assuming a linear relationship and superposition of the deviation of obtained 3D models, the theoretically worst-case scenario would be scanning the diaphysis transversally with the routine protocol using the Brilliance64 CT scanner and segmentation with the SAT algorithm, yielding a theoretical mean deviation of ~ 0.9 mm.

**Fig. 6:**
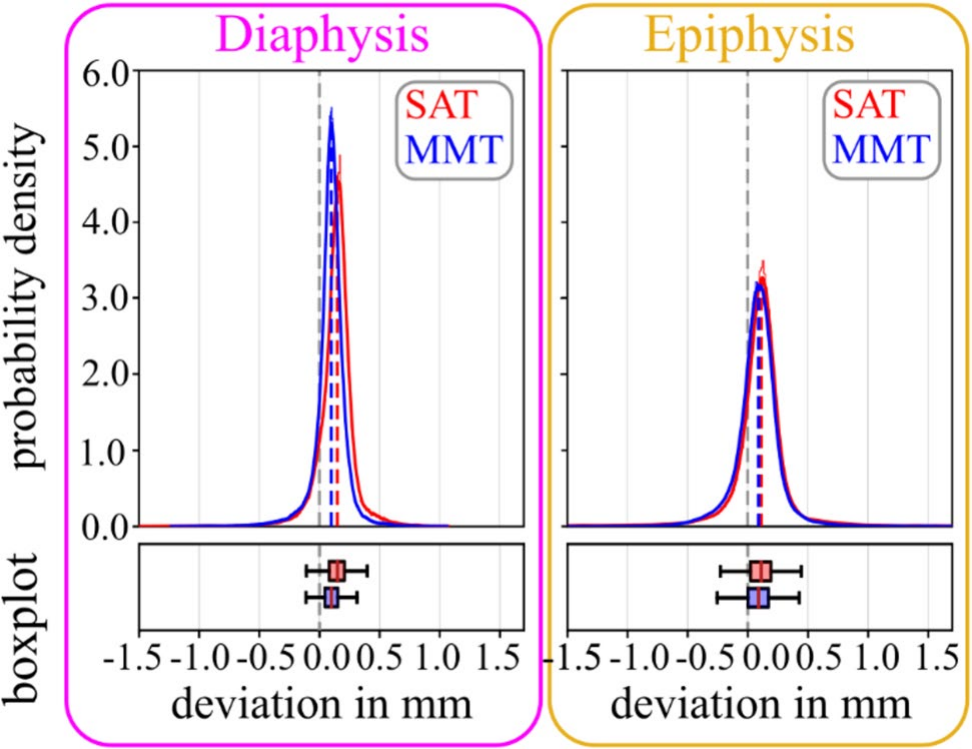
Deviation of 3D model dimensions, depending on segmentation algorithm (SAT: single automatic threshold vs. MMT: multi manual threshold), shown for diaphysis and epiphysis. Probability density functions are shown with histograms and corresponding boxplots (outliers are not plotted, for better visibility due to a large number of data points (∼100.000))

The manually determined threshold for cortical bone was 909 ± 44 HU for the diaphysis and 275 ± 58 HU for the epiphysis (for operator 1, baseline evaluation). The dimensions of 3D models based on the MMT algorithm deviated statistically significantly (*p* < 0.001) less than those based on the SAT algorithm (see Table 4; Fig. 6), whereby the difference was larger for the diaphyses. The average processing time for the SAT segmentation was 01:30 ± 00:29 min, compared to 09:25 ± 03:07 min for MMT.

**Table 4:** Deviation of 3D model dimensions, depending on segmentation algorithm (SAT: single automatic threshold and MMT: multi manual threshold, std: standard deviation, RMSE: root mean squared error, Min: minimum, Max: maximum, IQR: inter-quartile range; all units in mm)

	**Mean**	**Std**	**RMSE**	**Min**	**Max**	**Median**	**IQR**
**diaphysis**							
SAT	0.14	0.17	0.22	-2.02	1.07	0.15	0.13
MMT	0.09	0.14	0.16	-1.23	1.06	0.10	0.11
**epiphysis**							
SAT	0.10	0.30	0.32	-3.94	2.15	0.11	0.17
MMT	0.08	0.23	0.25	-3.63	2.44	0.09	0.17

Intra-operator reliability of the determined segmentation threshold was poor in terms of the ICC (< 0.40, both for epiphysis and diaphysis, respectively; see Table 5). Apparently, positioning of the line intensity profile was highly subjective over time. This effect was more pronounced for the epiphysis (ICC: 0.08) than for the diaphysis (ICC: 0.39). Similarly, inter-operator reliability was rated fair for the epiphysis (ICC: 0.43), and good for the diaphysis (ICC: 0.70; see Table 6). The SAT-based 3D models allowed for excellent intra-operator reliability with respect to the mean dimensional deviation (ICC: 0.91 for the epiphysis and 0.92 for the diaphysis). Accordingly, inter-operator reliability for the mean dimensional deviation of 3D models was excellent for the epiphysis (ICC: 0.78) and good for the diaphysis (ICC: 0.71). Hereby, the mean dimensional deviation across all operators was smaller than 0.15 mm for the diaphysis and 0.10 mm for the epiphysis.

**Table 5:** Intra-operator variability (operator 1) reported as intra-class correlation (ICC): mean and 95% confidence interval [lower and upper bound]

	**Segmentation threshold MMT**		**Signed average deviation 3D model SAT**	
	epiphysis	diaphysis	epiphysis	diaphysis
ICC mean	0.08	0.39	0.91	0.92
ICC 95% CI	[-1.03, 0.62]	[-0.61, 0.76]	[0.70, 0.97]	[0.77, 0.97]
p-value	0.417	0.153	< 0.001	< 0.001

**Table 6:** Inter-operator variability (5 operators) reported as intra-class correlation (ICC): mean and 95% confidence interval [lower and upper bound]

	**Segmentation threshold MMT**		**Signed mean deviation 3D model SAT**	
	epiphysis	diaphysis	epiphysis	diaphysis
ICC mean	0.43	0.70	0.78	0.72
ICC 95% CI	[0.05, 0.72]	[0.41, 0.87]	[0.58, 0.91]	[0.48, 0.87]
p-value	0.008	< 0.001	< 0.001	< 0.001

## Discussions

The current study investigated the effect of different CT technologies, methodologies and settings of image acquisition, image segmentation, and 3D model generation on dimensional accuracy of a specific bone fracture model. An established workflow was presented, whereby all mentioned sources of errors were analyzed separately. Mean absolute dimensional deviation ranged from 0.03 mm (epiphysis, NAEOTOM Alpha, optimized protocol) to 0.32 mm (diaphysis, Brilliance 64 scanner). These deviations can be queued in the lower half of previously reported values ranging from 0.13 to 0.62 mm [24, 30, 31, 43–45], however, this is the first study that reported the use of PCD-CT for bone fracture modelling. In a previous study on sheep femora [28], also a Brilliance 64 EID-CT was used to obtain 3D models with a global segmentation threshold, reporting a mean deviation of 0.24 mm. The slightly lower deviation in that study might be attributed to the higher tube voltage (140 kVp vs. 120 kVp), smaller slice spacing (0.50 mm vs. 0.67 mm), and different skeletal region (femora vs. radii). In the present study, the superior performance of the NAEOTOM Alpha PCD-CT and SOMATOM Force EID-CT was also reflected in a less frequent need to manually edit connected regions of bone tissue, e.g., of radius and carpal bones or skin. In general, the necessity for manual processing has been reported frequently [24, 43, 46] and highlights the necessary awareness of potential error sources generated already during image acquisition [47]. It should be noted that the maximum and minimum deviations across all CT scanners were mostly in the range of − 1.00 and 1.00 mm, indicating not only sufficient accuracy but also precision in representing the true geometric dimensions. Higher deviations were only visible in a few samples, caused by missed hole-like structures and overestimation of peak-like structures in the epiphysis (see inset Min-Max in Fig. 3). These issues are likely caused by the filling process during image processing, in combination with the partial loss of trabecular structures during the maceration process. Hence, it is assumed that these extreme values would be lower in clinical routine. Nevertheless, a visual evaluation by a radiologist or an experienced operator for the contour of the epiphysis in final models is recommended.

Previously, Van Eijnatten et al. [25] determined a significant effect of the selected CT scanner and scan protocol on 3D model accuracy of human skulls. However, they reported that the selection of the scanner is more important than the magnitude of tube voltage or tube current. Accordingly, Oka et al. [31] found no clinically significant effect on dimensional accuracy of 3D models (0.04 mm) when decreasing the tube current and increasing the pitch factor in a low-dosage protocol. Hence, both studies [25, 31] rated low-dosage protocols as overall superior for clinical routine due to their lower radiation exposure and clinically insignificant accuracy decrease. Based on these studies, in the present study, it was hypothesized that an optimized protocol (increased tube current and decreased pitch factor) would substantially increase the models’ accuracy. Indeed, applying the optimized protocol resulted in more accurate models for the NAEOTOM Alpha, SOMATOM Force, and Brilliance64 scanner, whereas no effect was observed for the Edge + scanner. Apparently, no general statement about the benefit of utilizing optimized scan protocols could be drawn across all investigated CT scanners. Further, the radiation dose ranged between 19.1 and 26.5 mGy (CTDI) for the optimized, compared to 4.4 to 5.3 mGy for the routine protocol. Therefore, one must question whether the enhanced accuracy ranging from 0.01 to 0.09 mm of mean deviation (20 to 33%, respectively, concerning the absolute deviation of the routine protocol) justifies a higher radiation exposure by a factor of 4–5. The observed differences between the CT scanners could be, on one hand, attributed to their year of introduction (2021 for NAEOTOM Alpha, 2017 for SOMATOM Force, and 2004 for Brilliance64), and on the other hand to the inherent technological advance, especially in terms of detector design and iterative reconstruction options. This leads to different dose efficiencies, spatial resolution limits, and consequently differences in image quality. Furthermore, the institutional standard protocols of the Medical University of Vienna were used for the routine acquisition and an optimized protocol to maximize the image quality. Therefore, a high-resolution (HR) mode was used for the NAEOTOM Alpha, SOMATOM Force, and the Brilliance64, as it was not available for the Edge+. This, to some extent, explains the increased model accuracy of the optimized scan protocol data sets for these three scanning systems. In the current study, all investigated scanners and scan protocols yielded 3D models with a mean dimensional deviation smaller than 0.33 mm, well below the required clinical threshold of 0.5 mm [31]. Thus, commonly used routine protocols with conventional CT scanners are likely sufficient to create accurate 3D models of bone and bone fractures.

Extremities (arm and leg) are preferentially scanned in approximated axial direction (as positioning is feasible) for improved image quality. However, under specific circumstances, e.g., difficult positioning of poly-trauma patients or in pre-clinical studies using anatomic specimens aiming to fit most specimens in single scan series, extremities are sometimes scanned perpendicular to their anatomical axes, e.g., in frontal or sagittal plane. In the present study, 3D models obtained from longitudinal scans were significantly more accurate than those from transversal scans. This effect was more pronounced in the diaphyses than in the epiphyses (see Table 3; Fig. 5). As such, several outliers of bone tissue spikes were visible in the diaphysis in transversal scans, causing not only an increased mean deviation, but also a larger right-skewness of the data. In the epiphysis, far fewer outliers of bone tissue spikes were present, and the dimensional standard deviation between the models was only minorly increased in transversal scans. Assumingly, the dimensional accuracy of the irregular shape of the epiphysis is only minorly dependent on the specimen orientation. In contrast, the cylindrical shape of the diaphysis changes dramatically, if observed from an orthogonal view. Accordingly, in a previous study [26], the model accuracy of the epiphysis of long bones (scanned longitudinally) was significantly affected by the selected CT slice thickness. The authors related this effect to the non-uniform shape of the epiphysis (rapid change of geometry in scan direction), whereas almost no effect was observed for the mainly uniform diaphysis (in axial direction). Accordingly, in the present study, the diaphysis indicated a rapid change of geometry normal to its axis, decreasing accuracy if scanned transversally.

In the literature [24], image processing and especially image segmentation have been described as the most crucial factors for 3D model accuracy. Comparably, image acquisition was reported to have a less prominent effect [24]. In the present study, models segmented with the manually determined threshold (MMT) for the epi- and diaphysis showed a higher dimensional accuracy, but in the range of only several micrometers, compared to models segmented with the global segmentation method (SAT). This finding is in accordance with a previous study [28], where an advanced multi-threshold approach also outperformed a single global threshold. In the present study, the multi-threshold segmentation performed better in the diaphysis than in the epiphysis, likely related to the thicker cortical bone. The manually determined mean lower thresholds for cortical bone were 275 ± 58 HU for the epiphysis and 909 ± 44 HU diaphysis (for operator 1), in comparison to the global lower threshold of 226 HU for bone. Hence, there was a higher effect of the segmentation algorithm in the diaphysis. Hereby, the identification of an adequate threshold for the thin cortical shell in the epiphysis is challenging. The applied algorithm assumes a clearly identifiable plateau of HU values for both bone and soft tissue in the line intensity profile [40]. The determined values of around 275 HU are thus likely an underestimation, a previously described issue with the segmentation of thin cortical shells [48]. The probable cause is the limited resolution of CT images and the relatively low thickness of the cortical shell. In the present study, a minimum of two adjacent voxels within the cortical bone shell was required to calculate the manual lower threshold. Still, the obtained values in the range of ~ 600 HU were significantly below the values of the diaphyseal cortical bone (~ 1800 HU). This discrepancy is likely related to the partial volume effect (PVE). Interestingly, the inter-operator variability in the study presented here indicated fair reliability (ICC: 0.43) for the lower segmentation threshold for the epiphysis, but good reliability (ICC: 0.73) for the diaphysis, using the proposed line intensity profile method. In contrast, the relative dimensional mean deviations across all operators were only 4.2 ± 3.5% for the diaphysis and 22 ± 21% for the epiphysis.

Previous studies [30, 43, 49] showed that the dimensional accuracy of bone models was highly dependent on selected CT image resolution, with a relative accuracy of 1/2 voxel, also for the RMSE, a measure for the dimensional precision. This is in line wiht the current study, where mean deviations and the RMSE (for models based on the routine scan protocol and the global segmentation) were also in the range of 1/4 to 1/2 voxel.

Using the latest detector technology in the PCD-CT scanner, 3D models with a superiorly higher accuracy < 0.05 mm were generated. Further, by optimizing the imaging with EID-CT scanners and enhanced segmentation parameters model inaccuracy was lowered to < 0.2 mm. Still, the question has to be raised what level of dimensional accuracy is necessary for a given application. In the present study, all investigated 3D models achieved mean deviations (< 0.32 mm) well below the reported thresholds of 0.5 mm [31] or 1.0 mm [28] for applications in orthopedic and trauma surgery. Combining the variables that caused the highest inaccuracies: scanning the diaphysis transversally with the Brilliance64, applying the routine protocol, and segmenting the image series with the SAT algorithms would still yield a mean deviation of ~ 0.9 mm and below the higher 1.0 mm threshold which is likely sufficient for all bone models.

The intra-operator reliability for 3D model design was excellent (ICC > 0.9) and the inter-operator reliability was good to excellent (ICC > 0.72). Despite the substantial relative variation especially for the epiphysis across the operators, the mean deviation of the dimensions across all operators was smaller than 0.16 mm. The SAT algorithm required an average of 1.5 min for the segmentation, compared to ~ 9.5 min for the MMT algorithm (close to a previously mentioned segmentation time of ∼11 min for 3D models for acetabular fracture surgery [15]). Additionally, around 1 to 3 min were required to fill the inside of the bone (irrespective of the used segmentation method). However, even ~ 10 min of image-processing time is only a small fraction of the total treatment time e.g., only the acetabular fracture surgery is estimated with ~ 3 h [15]. Consequently, more effective pre-operative planning might decrease operative time and result in a better clinical outcome, and thus, increase economic efficiency.

A major limitation of the current study was that only a single anatomical region was investigated. Further investigation is required to validate the obtained results in terms of anatomical location, bone quality with specific existing pathologies (e.g., osteoporosis), and age (children vs. adults or elderly) for different fractures (comminuted or compression fractures). Furthermore, only CAD 3D models were investigated in the present study. Hence, these models could also be 3D printed in a future study to determine the accuracy of these final, physical bone fracture models.

## Conclusions

3D models of the fractured distal radius could be obtained at sub-voxel accuracy (maximum mean deviation of 0.32 mm) for all investigated CT technologies/scanners, scan protocols, and segmentation algorithms. All investigated CT scanners and applied routine scanning protocols as well as a simple global thresholding algorithm for image segmentation yield sufficient dimensional accuracy and precision. However, even highly accurate 3D bone fracture models with a mean deviation < 0.05 mm can be obtained with PCD-CT based 3D models, which might be required for specific cases.

### Electronic Supplementary Material

Below is the link to the electronic supplementary material.

Supplementary Material 1

## Abbreviations

CAD: Compter-aided design
CT: Computed tomography
EID: Energy-integrating detector
PCD: Photon-counting detector
HU: Hounsfield unit
SAT: Single automatic threshold
MMT: Multi-manual threshold
ICC: Intra-class correlation
