# Supplementary figures and images for: Accuracy Analysis of 3D Bone Fracture Models: Effects of Computed Tomography (CT) Imaging and Image Segmentation
Source: J Imaging Inform Med. 2024 Mar 14;37(4):1889–901. doi: 10.1007/s10278-024-00998-y (PMC11300728; doi:10.1007/s10278-024-00998-y)

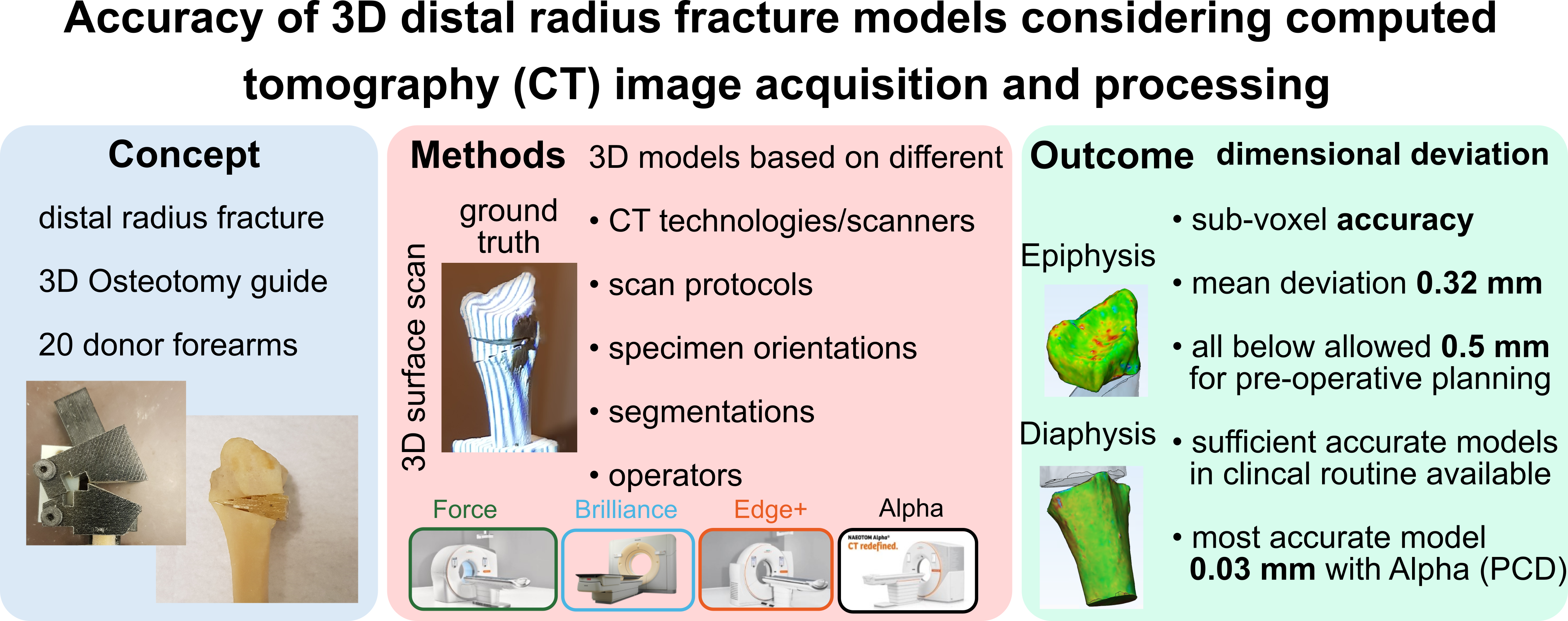

Supplement: Supplementary file 1 — Supplementary Material 1 [file 10278_2024_998_MOESM1_ESM.png]
